# Spontaneous and information-induced bursting activities in honeybee hives

**DOI:** 10.1038/s41598-023-37785-8

**Published:** 2023-07-07

**Authors:** Itsuki Doi, Weibing Deng, Takashi Ikegami

**Affiliations:** 1 Graduate School of Arts and Sciences, University of Tokyo, 3-8-1 Komaba, Meguro-ku, Tokyo, 153-8902 Japan; 2 Key Laboratory of Quark and Lepton Physics (MOE) and Institute of Particle Physics, Central China Normal University, Wuhan, 430079 China

**Keywords:** Animal behaviour, Behavioural ecology, Machine learning, Time series

## Abstract

Social entrainment is important for functioning of beehive organization. By analyzing a dataset of approximately 1000 honeybees (*Apis mellifera*) tracked in 5 trials, we discovered that honeybees exhibit synchronized activity (bursting behavior) in their locomotion. These bursts occurred spontaneously, potentially as a result of intrinsic bee interactions. The empirical data and simulations demonstrate that physical contact is one of the mechanisms for these bursts. We found that a subset of honeybees within a hive which become active before the peak of each burst, and we refer to these bees as "pioneer bees." Pioneer bees are not selected randomly, but rather, are linked to foraging behavior and waggle dancing, which may help spread external information in the hive. By using transfer entropy, we found that information flows from pioneer bees to non-pioneer bees, which suggest that the bursting behavior is caused by foraging behavior and spreading the information through the hive and promoting integrated group behavior among individuals.

## Introduction

It is well known that populations of agents, whether animate or inanimate, sometimes exhibit complex self-organization and emergence phenomena. An example of such self-organization in social organisms is known as "social entrainment/synchronization"^1^. The synchronization of group behavior is essential for a group's daily survival, as it enables the achievement of common goals, such as breeding, defense against predators, collective hunting, and energy conservation through gathering. Moreover, for a group to function as a superorganism beyond a mere collection of individuals, synchronization phenomena are likely indispensable. Groups of starlings and sardines are well-known examples of groups that function as superorganisms^2, 3^. Grouping reduces the susceptibility to predator attacks. Drosophila increases the probability of mating by synchronizing its circadian rhythm with that of the group^4–6^. Also, it is known that honey bees use waggle dance to notify the location of feeding area and to synchronize circadian clock within a hive is needed to transfer the information^7^.

In honeybees, numerous studies have been published regarding temporal synchronization among groups within the hive, particularly focusing on circadian rhythms. Southwick et al. (1987) suggested that bees use direct contact or vibrations, rather than volatile substances, to synchronize activity levels among individuals^8^. Korst et al. suggested that trophallaxis is a major form of communication mechanism among honeybees^9^. A recent study suggests that direct contact, for example via contact pheromones or tactile communication, is not necessary for the synchronization of circadian rhythm in the hive^10^. Furthermore, in some species of ants, which are also eusocial insects like honeybees, synchronization not only occurs on long timescales such as circadian rhythms but also in the form of self-organizing synchronization of periodic activities that occur in approximately 30-min cycles. The phenomenon was discovered in the 1990s, yet it is believed that these activity cycles are not functional but rather the inevitable outcome of interactions within social groups^11, 12^.

We can find parallels to the complex processes occurring within the brains and there are studies that discuss animal groups in comparison to cognitive science research^13, 14^. The brain is well-known for generating a large number of periodic/non-periodic synchronization activities, which are derived not only from responses to external stimuli but also from so-called "spontaneous activities" that regularly carry out firing activities without external inputs^15^, and this phenomenon is believed to play a role in stabilizing the performance of cortical circuits^16^.

In the past decade, tracking technologies have made significant progress in tracking individuals within biological populations, allowing for more detailed analysis of individual behavior within the group^17–20^. This consequently enables us to examine which individual (micro) characteristics contribute to self-organization at the population (macro) level. Gernat et al*.* (2018) developed a high-throughput automatic monitoring system of artificial honeybee hive of a single layer in a transparent cage. By attaching a "bCode" device (a custom matrix barcode) to the thorax of every individual bee in the hive, they succeeded in tracking each individual bee’s positions, speeds, and orientations using the recorded digital images. Gernat et al*.* used the tracking system to study bees’trophallaxis (mouth to mouth interaction to transfer food or chemicals) networks and calculate how often they communicated. They found that bees communication occurred in a temporally intermittent manner, which they refer to as bursts, much like human communication networks^17^.

In the present study, using the same large dataset as their research, we report on the global synchronous activity of locomotion, here we called ‘burst’, found in a population of European honeybees (*Apis mellifera)* and its relationship with individual activity. This collective burst is an extremely robust phenomenon observed under five different conditions in an artificial hive of approximately 1000 bees (Fig. S1). The burst occurs on a timescale smaller than the diurnal rhythm, such as circadian rhythm, and appears to be different from the oscillatory activity observed in the collective behavior of ants^11, 12^ in that the frequency of their bursts is Poissonian rather than periodic. Furthermore, this burst does not simply occur in response to external stimuli. The paper investigates the mechanism behind these bursts and analyzes whether the bees that initiate each burst called “pioneer bee (P)” (bees with increased activity prior to the burst) are randomly selected or if there are biological features that determine their selection. Moreover, based on the tracking data, we identified well-known honeybee biological behaviors, such as foraging, waggle dance, and dance following, and then examined to what extent these behaviors were performed by P. If the behavior of a small group of pioneer bees (micro), the bursting behavior, influences the behavior of the hive itself (macro), then a detailed study of pioneer bees of global bursts may provide an indicator for understanding the state of the hive.

## Results

### Entire tracking of bee behaviors

In this study, we used the entire tracking data, which were acquired by tracking all honeybees individually by placing an artificial hive in one planar layer (a normal honeybee hive has its planes in layers) with a population consisting of approximately 1000 adult worker bees and a single queen bee (a single cohort population). All data were provided by G. Robinson and his group from the University of Illinois Urbana-Champaign; their detailed data collection methods are reported in their paper^17^. To summarize briefly, they used one cohort hive consisting of approximately 1000 adult worker bees aged 1 day and one unrelated, naturally mated queen bee. As in other studies that have tracked individual social insects^18–20^, they successfully tracked the location and orientation of individual bees every second by placing a 2DQR barcode (bCode) on the thorax of each bee (Fig. S2). In July 2012 and July 2013, five trials were conducted, with each trial lasting approximately 1 week. The hive was located in a dark, quiet place, and its glass window was cleaned daily in the morning and at night to ensure a high detection rate of individual bees night and day throughout the experiment. The hive was connected to the outside by a door, which was closed for the first two days and then opened. Then, the worker bees could exit the hive and start exploring the outside and foraging. All individual bees were identified by the bCode and tracked over 7 days. As there were no larvae or pupae in this artificial hive, no caretaking role was observed.

### Characteristics of bursts

**Figure 1:**
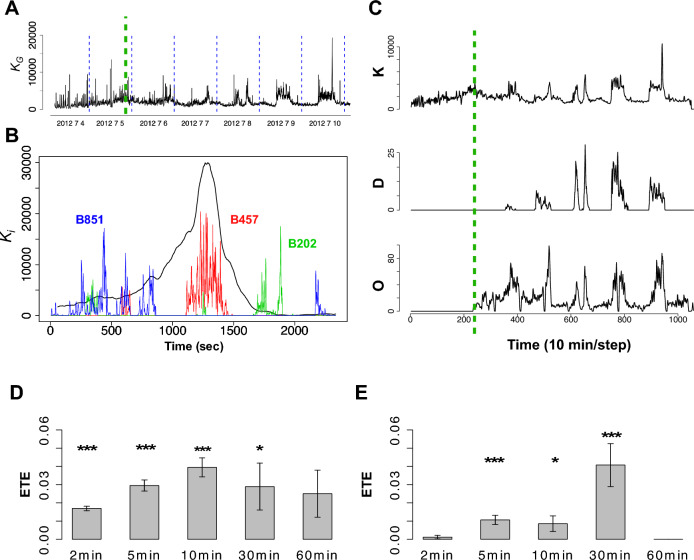
The individual and collective activity of a bee. (**A**) An example of the time series of ${K}_{G}(t)$ (Trial 1201). The vertical dotted blue lines indicate the midnight of each day. The time the entrance was opened is indicated by the green dotted line. (**B**) An example of ${K}_{i}\left(t\right)$ and ${K}_{G}(t)$ in an endogenous bursting region. The black curve describes ${K}_{G}(t)$, and the colored curves describe ${K}_{i}\left(t\right)$ (for individuals with the bCodes B457, B202, and B851). The amplitude of ${K}_{G}\left(t\right)$ was rescaled to compare with ${K}_{i}\left(t\right)$*.* (**C**) Three observation quantities of the hive state in Trial 1201. One time step is 10 min. K represents the overall time series of ${K}_{G}\left(t\right)$, D represents the time series of the number of bees which performed waggle dance within each time step, and O is time series of the number of *out-of-hive* bees within each time step. (**D**) Effective transfer entropy (ETE) from the time series of D to K (${ETE}_{D\to K} -$ ${ETE}_{K\to D})$. (**E**) Effective transfer entropy (ETE) from the time series of K to O ${(ETE}_{K\to \mathrm{O }}- {ETE}_{O\to K })$. In D and E, ***, **, and * indicate significant difference between ${{TE}}_{{X}\to {Y}}$ and ${{TE}}_{{{X}}_{{shuffle}}\to {Y}}$ on the 0.1%, 1%, and 5% significance levels, respectively.

The activity of an individual bee (i) can be measured as its kinetic energy (K), i.e., a square of velocity. The hive activity level ${K}_{G}(t)$ at time t is defined by averaging the individual kinetic energies. By tracking the individual bee behavior, we defined and measured the activity of the individual bee (i) using the kinetic energy as follows:

$${K}_{i}\left(t\right)=\Delta \hspace{0.17em}{x}^{2}+\Delta \hspace{0.17em}{y}^{2}\hspace{0.25em},$$

where $\Delta \hspace{0.17em}x$ and $\Delta \hspace{0.17em}y$ denote the respective displacements of the *x* and *y* coordinates of each bee (i) per second. The overall hive activity level ${K}_{G}\left(t\right)$ was defined by averaging over the individual kinetic energy as follows:

$${K}_{G}\left(t\right)=\frac{1}{n}{\sum }_{i=1}^{n}{K}_{i}\left(t\right),$$

which quantifies the global activities of the hive (*n* is the total number of the bee). When the bursts are synchronized in phase, ${K}_{G}(t)$ should increase. In fact, ${K}_{G}(t)$ was found to increase rapidly in time and then relax, which we identified as a bursting phenomenon (Fig. 1A,B). For the mechanical detection of bursts, we applied a commonly used algorithm known as Kleinberg’s burst detection method^21^. An advantage of this method is that bursts are systematically identified based on a variable baseline of activity. For each beehive, the experiment lasts for seven days. The number of bursts detected in each trial was as follows: 71 in Trial 1201; 48 in Trial 1202; 58 in Trial 1203; 69 in Trial 1301; and 66 in Trial 1302.

During the bursting phase, we observed that the excited honeybees moved around in the hive and frequently collided with other bees (see supplement). Initially, the door of the hive was closed, and after two days, the door was opened to allow the bees to go outside. The bursts occurred spontaneously irrespective of the opening and closing of the door. There are also other bursts caused by artificial disturbances (i.e., caused by the act of cleaning the glass of the hive case). The features of the artificial bursts caused by glass cleaning are sudden increases in ${K}_{G}(t)$. The spontaneous bursts and artificial bursts were distinguishable because their timing was recorded during the experiment.

Throughout the trial, the percentage of bees active with above-average kinetic energy at the peak of each burst was 50.5% (± 15.5%). Actually, when ${K}_{G}(t)$ increases, the number of bees committing to ${K}_{G}(t)$ also increases. Also, the distributions of inter-burst intervals among every trial obeyed power law like distribution which was fitted with maximum likelihood methods as introduced by Clauset^22^ (p-value > 0.647). This result suggests that these bursts are different from the periodic activity cycles observed in e.g. ants^11, 12^.

### KDO measures

Initially, we examined globally how the global burst correlates with other behavior features of the bees. In addition to the aforementioned time series of activity level (K), we identified the time series of the number of *out-of-hive* bees within each time step (O) and the time series of the number of the individuals that performed waggle dance, which is communication method to convey information about the location of food sources to other bees, within each time step (D) (Fig. 1C).

To examine the relationship of influence between these K, D, and O time series, we calculated effective transfer entropy (ETE)^23^ between them. ETE is a metric used to quantify the directional flow of information between two interacting systems or variables, which is an improvement of the original transfer entropy (TE) developed originally by Schreiber^24^. It helps in understanding causal relationships and dependencies between systems by measuring the degree to which the past state of a system can be used to predict the future state of another system In other words, the difference assesses the strength and direction of information flow between systems which one is in the upper stream and which one is in the downstream of the information flow: a positive ${ETE}_{X\to Y}-{ETE}_{Y\to X}$ implies information flow from X to Y and a negative ${ETE}_{X\to Y}-{ETE}_{Y\to X}$ implies information flow from Y to X. (see “Methods” for details).

ETE is sensitive to the time bin of data sampling. In this study, we calculated the ETE between the time series of K, D, and O while varying the time bin size, ranging from a few minutes to an hour (2, 5, 10, 30, and 60 min). K represents the overall time series of ${K}_{G}(t)$, D represents the time series of the number of bees which performed waggle dance within each time step, and O is time series of the number of *out-of-hive* bees within each time step.

As shown in Fig. 1D,E, there was the maximum value of the difference between ${ETE}_{D\to K}$ and ${ETE}_{K\to D}$ (i.e., ${ETE}_{D\to K} - {ETE}_{K\to D}$ at around a 10 min time bin. On the other hand, the maximum value of the difference between ${ETE}_{K\to O}$ and ${ETE}_{O\to K}$ was at 30 min time bin. Namely, there were maximum information flows from D to K on a timescale of 10 min, and there were maximum information flows from K to O on a timescale of around 30 min. These results suggest that there is a temporal order in which the burst occurs after the dance takes place and then the bees fly out from the hive.

### Classifying pioneer bees

As we approximately determined the temporal ordering of K, D, and O, we investigated each burst with respect to the activities of the individual bees. Here, under the hypothesis that bursts are triggered by bees whose activity increases first, we extracted these trigger bees and investigated their characteristics.

We tested the above hypothesis using experimental data and an agent based model. As a results, we found that physical contact could be a contributing factor to spontaneous bursts, both from experimental analysis and agent-based model simulations (see Supplementary Information for details). These results suggest that the bees becoming active prior to a spontaneous burst may trigger the burst.

**Figure 2:**
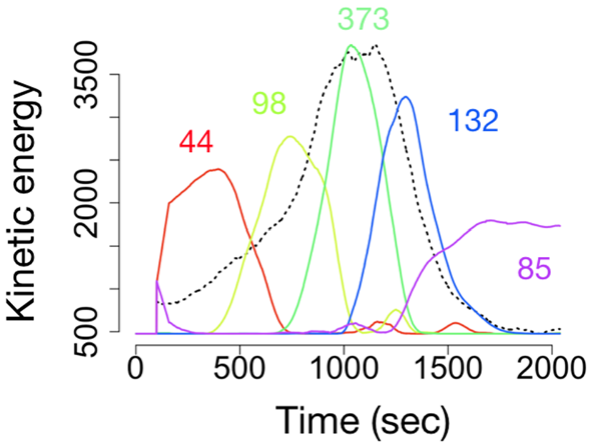
An example of using NMF to decompose the burst pattern into groups. An example of using NMF to decompose the burst pattern into groups. Each number on the graph indicates the number of individuals belonging to that particular group. The black dotted lines indicate global kinetic energy ${K}_{G}(t)$, and the colored lines represent the decomposed bases. The numbers describe the number of bees categorized in each base. In this case, the first group in time (the one marked as 44 in the figure) represents the pioneer bee.

To extract the subset of bees which activate first before the global burst, we used a technique known as nonnegative matrix factorization (NMF) to dissect bursts^25, 26^ (Fig. 2). This technique does not decompose bursts into periodic modes like the Fourier transform, but it does to a multiplication of two matrices. As this expression is not uniquely determined, we selected a best rank order of matrix to minimize the Kullback–Leibler (KL) divergence of the original and re-expressed the matrix.

To explain NMF in more detail, the matrix from each burst expresses the activity of all the bees sorted by its bCode (n) at time (t), and the matrix elements are the kinetic energy $$K(n,t)$$ of the bees. The matrix $$K(n,t)$$ is expressed by the product of two new matrices, $$W$$ and $$M$$, and the group (m) of bees with high activity at each time is obtained from $$K(n,t)$$ = $$W(n,m)$$$$M(m,t)$$. When $$M(m,t)$$ is plotted along the time axis, we can identify the individual groups with higher activity in the burst period. The contribution of an individual to each group is represented by $$W(n,m)$$. The optimal size *m* is determined by the KL divergence (see Methods section for details). Here, we named “pioneer bees” ($$\mathrm{P}_\mathrm{b}$$), which is a particularly active subset at the very beginning of each burst (the suffix "b" expresses Burst ID).

Various individuals are involved in each burst, but it is necessary to investigate whether $\mathrm{P}_\mathrm{b}$ is randomly determined for each burst or is determined as a social role.

In this analysis, we identified well-known honeybee biological behaviors, such as forager (F), waggle dancer (W), and dance follower (DF), using the tracking data. We then examined to what extent these behaviors were performed by $\mathrm{P}_\mathrm{b}$. It is crucial to note that the tracking data analyzed in this study were not obtained from a mature hive in the field, but from a single cohort colony composed of newly hatched bees. Consequently, roles within a single cohort colony may not be as firmly established as they would be in a fully matured hive (e. g., it is generally believed that bees become foragers about three weeks after hatching). There is no guarantee that a bee performing foraging in the first half of the experiment, yet will continue to do so in the latter half. In other words, the tracking data examined here derived from a hive in the midst of its maturation process, rather than a matured hive. Therefore, instead of employing an approach such as labeling a bee as a forager after it has performed foraging behavior at least once, we deemed it appropriate to redefine foraging, dancing, and dance following for each burst event, just as $\mathrm{P}_\mathrm{b}$ is defined for each burst. Namely, we determined $\mathrm{F}_\mathrm{b}$, $\mathrm{W}_\mathrm{b}$, and $\mathrm{DF}_\mathrm{b}$ for each burst in addition to ${\mathrm{P}}_{\mathrm{b}}$.

Since dance and dance following have well-known characteristic behavior patterns and are events that take place in the hive, it is possible to examine to what extent $\mathrm{P}_{\mathrm{b}}$ performed dance and dance following using our tracking data. On the other hand, foraging behavior is a behavior that takes place outside the hive, and our data did not allow us to determine whether the bees returned to the hive with food (e. g., pollen on their legs), so it was necessary to determine some evaluation criteria. We used two criteria for ${\mathrm{F}}_{\mathrm{b}}$: first, we defined bees that returned to the hive 10 min before the onset of the burst. As we mentioned before, we found that bursts are triggered approximately 10 min after dance events by using ETE between K and D (Fig. 1D). Since dancing is generally performed by bees returning from outside, this places the peak of bursts around more than 10 min after their return. Therefore, we have designated the bee returning from outside around 10 min before the peak of the burst as a ${\mathrm{F}}_{\mathrm{b}}$. The second criterion was to exclude "orientation flights," which are exploratory activities^27, 28^. We identified the first day of foraging as orientation flights by disregarding the initial six flights outside the door^29^.

**Figure 3:**
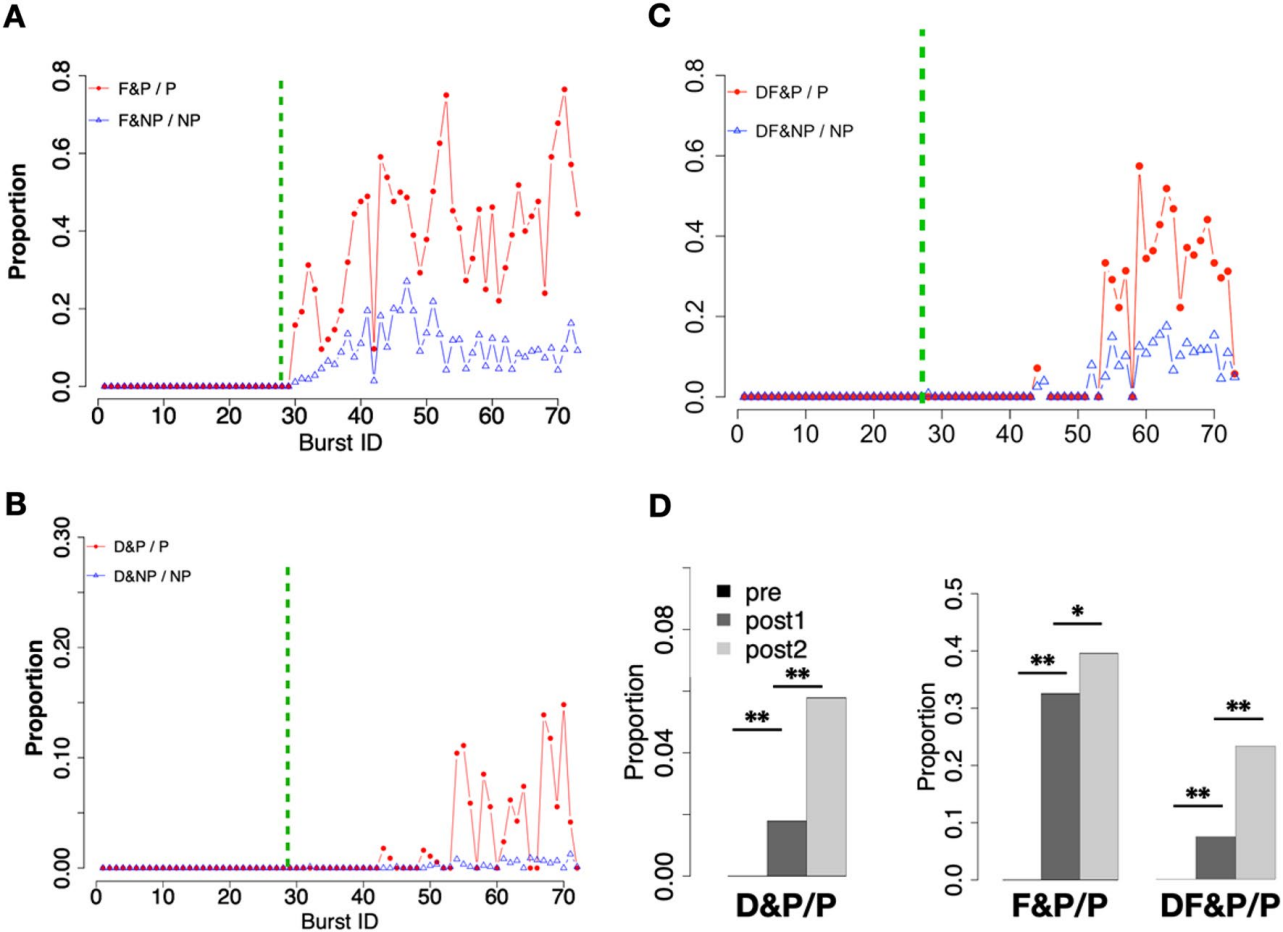
Time development of a honeybee hive. (**A**) The proportion of foragers (${\mathrm{F}}_{\mathrm{b}})$ among the pioneer (${\mathrm{P}}_{\mathrm{b}})$ and nonpioneer bees (${\mathrm{NP}}_{\mathrm{b}})$ in each burst. The time the entrance was opened is indicated by the green dotted line in Trial 1201. (**B**) The proportion of dancers (${\mathrm{W}}_{\mathrm{b}})$ among the pioneer and nonpioneer bees in each burst in Trial 1201. (**C**) The proportion of dance followers (${\mathrm{DF}}_{\mathrm{b}})$ among the pioneer and nonpioneer bees in each burst in Trial 1201. (**D**) The time development of the average proportion of all the trials: (left) the development of the average proportion of dancers among the pioneer and nonpioneer bees; (middle) the development of the average proportion of foragers among the pioneer and nonpioneer bees; and (right) the development of the average proportion of dance followers among the pioneer and nonpioneer bees. “Pre” refers to before the hive entrance was opened, “post 1” refers to days 1–3 after the hive entrance was opened, and “post 2” refers to day 4 to the last day after the hive entrance was opened. The p value was determined using the Brunner-Munzel test. ∗∗ p < 0.01, ∗ p < 0.1.

First, we examined the relationship between ${\mathrm{F}}_{\mathrm{b}}$ and ${\mathrm{P}}_{\mathrm{b}}$. Hereafter we will focus on Trial 1201 as representative of the experimental results. On the first day after opening the door, roughly 40% of the ${\mathrm{P}}_{\mathrm{b}}$ was ${\mathrm{F}}_{\mathrm{b}}$ (i.e., ${\mathrm{F}}_{\mathrm{b}}\wedge \mathrm{P}_{\mathrm{b}}/\mathrm{P}_{\mathrm{b}})$. Three days after opening the door, ${\mathrm{F}}_{\mathrm{b}}\wedge \mathrm{P}_{\mathrm{b}}/\mathrm{P}_{\mathrm{b}}$ was computed as 40.1% ± 3.89% on average (Fig. 3A). If considering a bee that has performed foraging at least once throughout the experiment to be ${\mathrm{F}}_{\mathrm{trial}}$, the percentage of ${\mathrm{F}}_{\mathrm{trial}}$ that also became a pioneer bee at least once (i.e., ${\mathrm{F}}_{\mathrm{trial}}\wedge \mathrm{P}_{\mathrm{trial}}/\mathrm{F}_{\mathrm{trial}})$ became 87.8%.

Next, the relationship between ${\mathrm{W}}_{\mathrm{b}}$ and ${\mathrm{P}}_{\mathrm{b}}$ was examined. On the first day of opening the door, ${\mathrm{W}}_{\mathrm{b}}$ was not included in ${\mathrm{P}}_{\mathrm{b}}$ because very little dance events occurred in this phase, but three days after opening the door, the percentage of ${\mathrm{P}}_{\mathrm{b}}$ that showed ${\mathrm{W}}_{\mathrm{b}}$ (i.e., ${\mathrm{W}}_{\mathrm{b}}\wedge \mathrm{P}_{\mathrm{b}}/\mathrm{P}_{\mathrm{b}})$ was nearly 10% (Fig. 3A). Considering the bees that exhibited waggle dance at least once throughout the experiment as ${\mathrm{W}}_{\mathrm{trial}}$, the percentage of ${\mathrm{W}}_{\mathrm{trial}}$ that exhibited P at least once (${\mathrm{W}}_{\mathrm{trial}}\wedge \mathrm{P}_{\mathrm{trial}}/{\mathrm{W}}_{\mathrm{trial}})$ became 89.6%.

Finally, we examined the relationship between ${\mathrm{DF}}_{\mathrm{b}}$ and ${\mathrm{P}}_{\mathrm{b}}$. DF are literally bees that observe dancing bees. DF have been known as agents collecting information about food locations by watching the dance^30–32^. Here, we defined a DF as an individual that is close enough to the dancer to turn its face toward the dancer. The percentage of ${\mathrm{P}}_{\mathrm{b}}$ that were also ${\mathrm{DF}}_{\mathrm{b}}$ after the third day of the door opening was 35.0% ± 4.31% on average (Fig. 3C), however, ${\mathrm{DF}}_{\mathrm{trial}}\wedge \mathrm{P}_{\mathrm{trial}}/\mathrm{DF}_{\mathrm{trial}}$ became 84.2% throughout a week.

**Figure 4:**
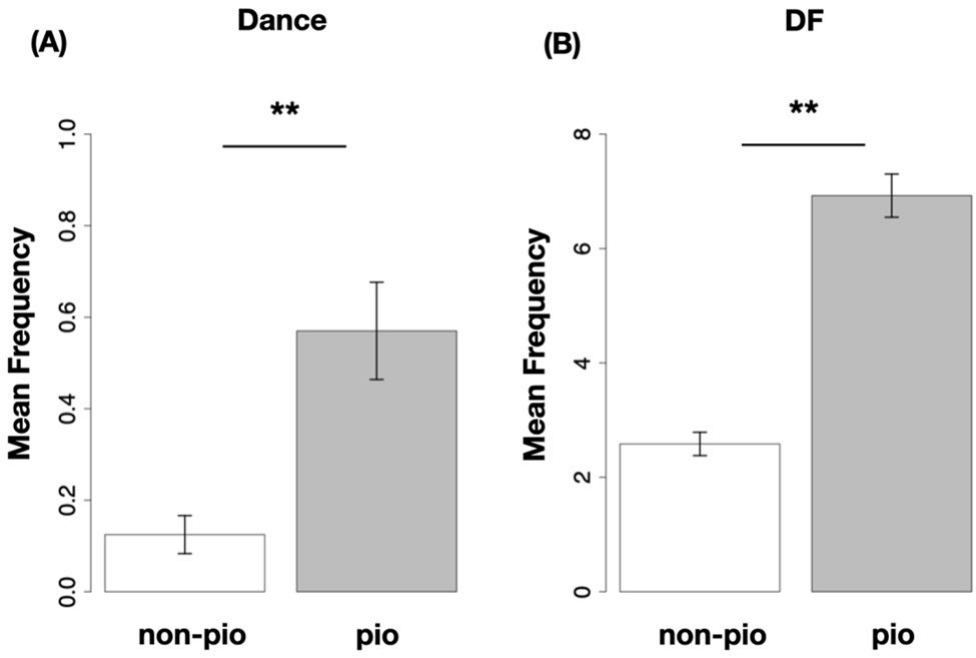
Waggle dancer, dance follower vs. pioneer bees. Bees that became pioneer bees at least once throughout the experiment tend to spend more frequently doing both the waggle dance (**A**) and the dance following (**B**) than non-pioneer bees (bees that never became pioneers throughout the experiment). The p value was determined using the Brunner–Munzel test. ∗  ∗ p < 0.05.

In summary, when the door was opened, the bees went out to forage, and on average across all trials, approximately 30% of the returning bees became pioneer bees. A few days after the door was opened, dancers and dance followers emerged, and most bursts were accompanied by dancers and dance followers. These trends were also observed in all five trials (Fig. 3D). In addition, by calculating the mean frequency of dancing and dance following for bees that became a pioneer bee at least once ($\mathrm{P}_{\mathrm{trial}})$ and for bees that did not become a pioneer bee at all during the experimental period ($\mathrm{NP}_{\mathrm{trial}})$, we found that bees that experienced “pioneering” tended to perform more frequently both waggle dances and dance follows (Fig. 4A,B).

Where the rest of the pioneer bee come from? We distinguished “foraged pioneer bees” (${\mathrm{FP}}_{\mathrm{b}}$ bees, i.e. ${\mathrm{F}}_{\mathrm{b}}\wedge {\mathrm{P}}_{\mathrm{b}}$) that foraged and returned to become a pioneer bee and “non-foraged pioneer bees” (${\mathrm{NFP}}_{\mathrm{b}}$ bees, i.e., ${\mathrm{NF}}_{\mathrm{b}}\wedge {\mathrm{P}}_{\mathrm{b}}$) that were a pioneer bee but did not leave a hive. FP and NFP bees spatially spread differently in the hive (Fig. S10). Both FP and NFP bees came near the door, but at the end of the post-phase (at around day 7), FP bees were still near the entrance, whereas NFP bees moved to the middle of the nest. Moreover, by calculating the kinetic energy KE for each of FP, NFP, and non-pioneer bees (NP), and the transfer entropy (ETE) among their time series (FP to NFP, FP to NP, NFP to NP and opposite directions), we found that there is a tendency for information flow from FP to both NFP and NP, and from NFP to NP. There is little or no information flow in the opposite direction (e.g., from NP to FP) (Fig.S11). In terms of information spread, these results suggested that FP bees brought information from the outside into the nest, and NFP bees were the nonforaging bees that cause the global burst spreading information throughout the hive.

### Temporal changes in bursting

Finally, to characterize the burst in terms of pioneer bees, we investigated the similarities between the combinations of pioneer bees in each burst.

First, each burst was indexed by the individual members in ${\mathrm{P}}_{\mathrm{b}}$. Then, using this index (i. e. a pioneer bee matrix), the distance between bursts was determined, and each burst was projected onto a two-dimensional plane (this method is known as multidimensional scaling [MDS])^33^. In this two-dimensional space, the distance between the points shows the similarity of the bursts with reference to the constituent members of the pioneer bees in each burst. Therefore, if the same bees are pioneer bees during different bursts, then these bursts are plotted near each other; otherwise, they are plotted further apart.

**Figure 5:**
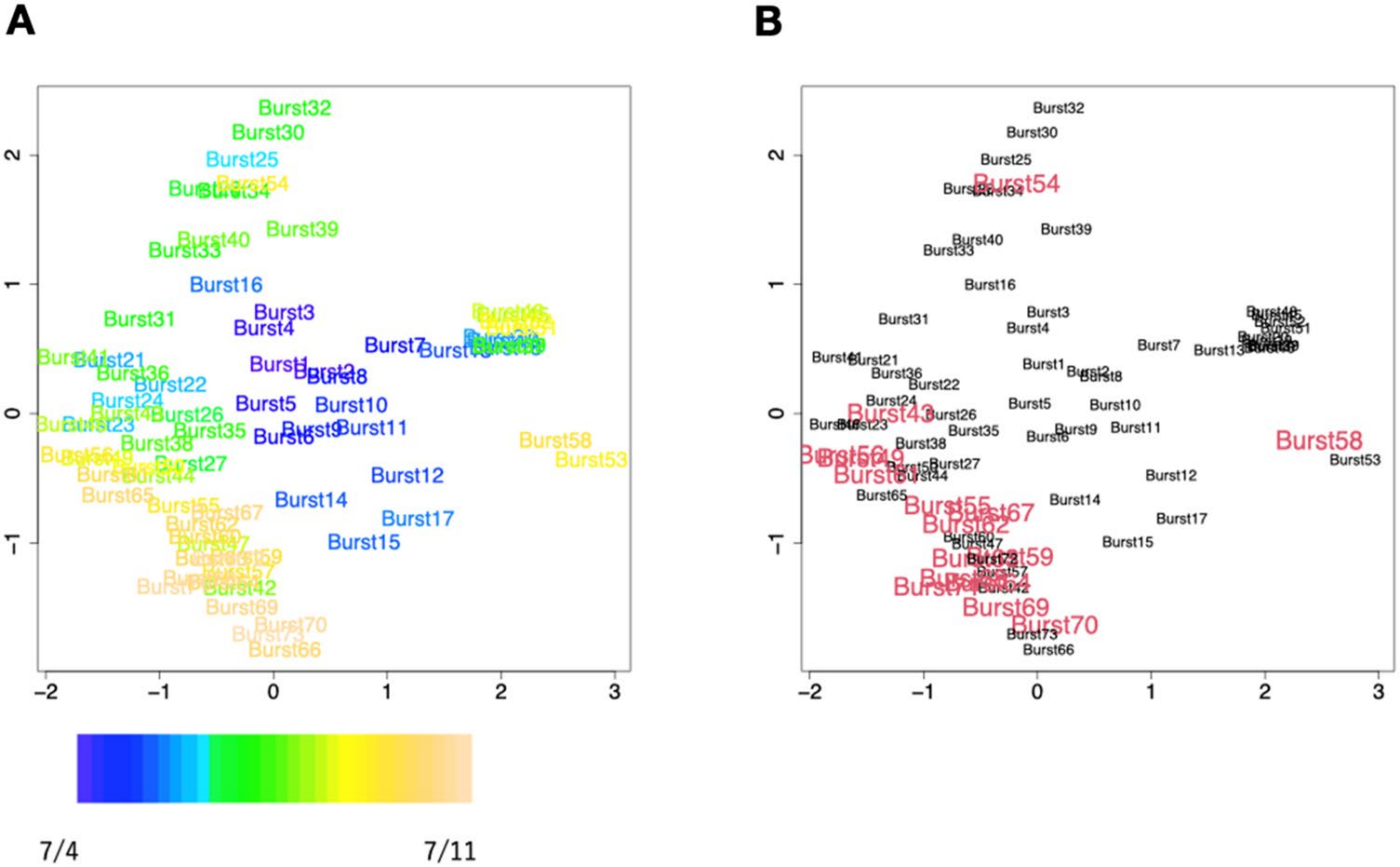
Classifying bursts according to individual pioneer bees using MDS and how bursts evolve over time (Trial 1201). (**A**) Time transition of the bursts in terms of the member of pioneer bees. The colors describe the transition of the date when the burst occurred (from blue [July 4] to beige [July 11]). The numbers associated with each burst form an ordered index, from 1 to 71. (**B**) Classifying bursts according to those preceded by waggle dancing (red) or no dancing. The position of each burst is the same as in Fig. 6A.

 As shown in Fig. 5A, comparison of the bursts before and after the door was opened. Before the door was opened, it is represented in blue text, and that after the door was opened is represented in green to yellow text; We see that after the door was opened, the burst were gradually converging in the MDS space. These results suggest that the member of pioneer bees are not always the same and that they do not seem to be randomly selected, but rather seem to change overtime. Furthermore, when focusing on bursts containing the dancers in pioneer bees (i.e., ${\mathrm{W}}_{\mathrm{b}}$ in ${\mathrm{P}}_{\mathrm{b}}$), these bursts seemed to aggregate within the MDS space (Fig. 5B), and a similar tendency was observed in other trials as well.

**Figure 6:**
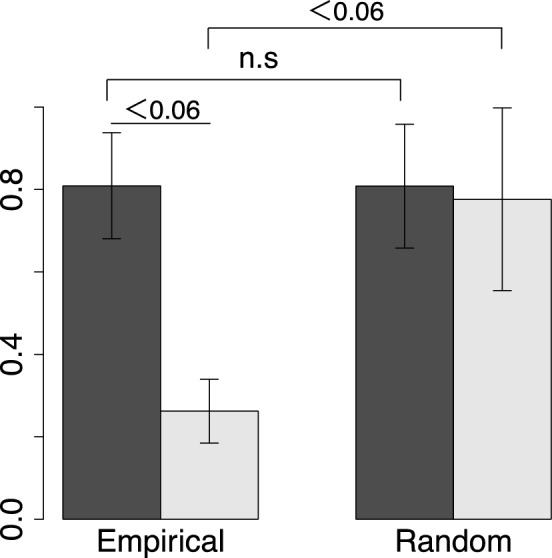
Statistical analysis of the MDS space. The variance within the MDS space between the bursts in the last two days (light gray) and the bursts in the first five days (dark gray). On the right hand side, the bar graph shows the variances in MDS space randomly (500 times) selecting the pioneer bees in each burst while keeping the number of bees the same as in the real data. Error bars indicate the standard error in values between trials. The p value was determined using the Brunner–Munzel test.

By examining the variances between bursts in the last two days of the experiment, and earlier bursts (in the first five days) across all trials in the MDS space, we statistically examined the convergence of the members of ${\mathrm{P}}_{\mathrm{b}}$. (Fig. 6). As a control, we calculated the variance within the MDS space after randomly selecting ${\mathrm{P}}_{\mathrm{b}}$ while keeping the number of ${\mathrm{P}}_{\mathrm{b}}$ the same as in the real data. From this analysis, we found that the member of ${\mathrm{P}}_{\mathrm{b}}$ in the early bursts after the hive opening shows no significant difference from random selection. However, the member of ${\mathrm{P}}_{\mathrm{b}}$ in the later bursts (6  days after the experiment started) is not randomly organized. These results suggest that ${\mathrm{P}}_{\mathrm{b}}$ in the later bursts of the experiment gradually converges as time passes. Since our experimental data is only available for seven days, it is not possible to examine how ${\mathrm{P}}_{\mathrm{b}}$ changes beyond seven days. Furthermore, the bee colony from which we collected our data was a single-cohort colony, a unique condition that wouldn't occur in the wild. Notably, in a wild hive, new adult bees continuously emerge, and foragers frequently perish due to various accidents, causing regular turnover among hive members.

Under the single cohort colony condition, we expect that ${\mathrm{P}}_{\mathrm{b}}$ will further converge to a particular member over time, however, in actual wild hives, we speculate that they likely align with certain roles such as foragers, dancers, and dance followers rather than pioneer bees converging on specific individuals.

## Discussion

In this study, we investigated the bursting behavior of honeybees, a form of social synchronization in locomotion activities, by analyzing the tracking data of approximately 1,000 honeybees across five trials. These bursts were predominantly spontaneous, and that these bursts in honeybees occurred irrespective of the hive entrance being open or closed. We aimed to understand the underlying mechanisms that drive these bursts, their relationship with the roles of bees within the hive, and the potential implications for the overall functioning of the hive.

Initially, to examine global bursts, we calculated information flows between the time series of bursts (i.e., global kinetic energy, ${K}_{G}(t)$), dance events and the number of bees outside the hive (O). Our findings indicate that events transpire in the following sequence after a dance event is confirmed: dance, burst, and departure from the hive.

Furthermore, we demonstrated that physical contact could be a contributing factor to spontaneous bursts, both from experimental analysis and agent-based model simulations (see Supplementary Information for details). These results suggest that bees becoming active prior to a spontaneous burst may trigger the burst. We designated these bees as "pioneer bees"(P) and employed non-negative factorization to identify them and investigated the characteristics of P. We examined each burst from the perspective of the members of P and the condition (for example, the entrance was opened or not) of the hive.

During the phase before the hive entrance opened, bees had no direct interaction with the external environment, yet spontaneous burst events still occurred. At this stage, forager (F), waggle dancer (W), and dance follower (DF) did not exist. We speculate that these bursts may resemble the spontaneous periodic activity cycle observed by Cole in isolated ant populations in 1991^12^.

The 1–2 days after the entrance opened, approximately 30% of the ${\mathrm{P}}_{\mathrm{b}}$ performed foraging before the burst. However, based on the multidimensional scaling (MDS) analysis results, it seemed that the selection of the members of P at this stage was slightly organized compared to random selection, but no significant difference was observed. When we examined the spatial distribution of foraged P (FP bees, i.e., ${\mathrm{F}}_{\mathrm{b}}\wedge {\mathrm{P}}_{\mathrm{b}}$) and non-foraged P (NFP) inside the hive, both were aggregated near the entrance. From these findings, we hypothesize that the burst at this stage was not so much ordered as it was a “spontaneous commotion” resulting from the hive being opened to the outside.

The 3–4 days after the entrance opened, we observe dancing and following behaviors, thus P members included not only F but also W and DF. Multidimensional scaling (MDS) analysis results revealed that P members were somewhat organized, unlike the bursts in a closed case. Moreover, when examining the spatial distribution inside the hive, FP was located near the entrance, as before, while NFP was situated toward the back of the hive. When assessing the information flow between FP, NFP, and non-pioneer (NP) using a method called transfer entropy, we discovered a significant flow of information from FP to NFP (Note that there was no significant information flow between FP and NFP during the 1–2 days after the entrance opened) (Fig. S11).

The tracking data used for this analysis only covers the inside of the hive, so it cannot demonstrate whether bees which performed foraging or dance actually found the feeding site and brought this information back to the hive. However, considering that the waggle dance is known to convey information about external feeding sites to bees inside the hive, and taking into account the results thus far including the temporal correlation between the dance event and the burst, it is plausible that bursts are utilized to efficiently convey such information.

Furthermore, considering the information flow between FP and NFP, it is suggested that the commotion caused by returning bees near the entrance of the hive spreads the burst to the interior of the hive. In other words, we may argue that these bursts were induced by “external information*”* rather than purely self-excited. Note that not all bursts following the appearance of W are necessarily information-induced. Figure 3B showed that there were bursts which P did not contain W even after their appearance inside the hive. Even after bursts with W occur, there may still be spontaneous bursts that are not apparently “information induced”.

From the perspective of regulating the division of labor, addressing the question of how workers acquire information on colony requirements, Robinson (1992) proposed a hypothesis that sampling behavior (i.e., exploration behavior of states in the hive) with social interaction may be facilitated by worker activity synchrony observed in ant colonies^34^. Furthermore, “mature hive” may indicate that the activities of individual bees are constrained by the entire hive (downward causation)^35^, and as proposed by E.O. Wilson et al*.* it would imply role differentiation^36^. Burst phenomena in bee colonies may also promote or regulate division of labor; however, to prove this, it is necessary to prepare a hive in which bursts are intentionally suppressed and compare it with a normal hive. Additionally, since this study only has data for the first seven days after bees were born, analyzing longer tracking data can provide more precise insights into the hive's differentiation.

Lastly, the question of which indicators can be used as measures of the state of populations (hives) is an attractive challenge in the research of collective behavior. The pioneer bees' behavior and composition may serve as indicators of the state of the hive, providing valuable insights into the dynamics of collective behavior in honeybee colonies.

## Methods

### Data

All data were provided by G. Robinson and his group from the University of Illinois at Urbana-Champaign; their detailed data collection methods are reported in their paper (17). To summarize briefly, they used a single-cohort hive consisting of approximately 1000 adult worker bees aged 1 day and one unrelated, naturally mated queen. This type of colony is commonly used in experiments analyzing the division of labor to control the effect of age and, instead, focus on the behavioral development of worker bees as a function of their genetics and social interactions^37^.

Similar to other approaches for tracking insects^18–20^, their method used custom 2D QR-barcode devices, known as *bCode*. These devices were attached to the thoraxes of individual bees and provided sequences of digital images that enabled reliable identification and tracking of every individual in the hive. The digital images were converted into data with coordinates (x, y) and the orientation of each bee every second, with x ranging from 0 to 6576 and y ranging from 0 to 4384. The hive was installed vertically in a dark room with a consistent temperature (approximately 35 °C) and humidity, and it was connected to the outside through a tunnel, whose entrance was closed until 2 or 3 days after the start of the observation and then opened to allow the workers to exit the hive and begin foraging (Fig. S1). The bees produced sufficient honey to feed the entire hive for the duration of the experiment, with sufficient bee nutrition for 2 days. Some bees could not be tracked because they died and because of sublations of *bCode*. The researchers removed the bees that could not be tracked from the tracking data before opening the entrance. The software could identify the bees in 94% of all cases (determined by a manual analysis of 60 images), with an error rate of 1.4% (determined by a manual analysis of 5000 detected barcodes). There were five separate trials (five different hives) of the experiments over July 2012–2013 (Table S1).

### Detection of a “forager”

After opening the entrance, bees often went outside. It is well known that bees go outside for “foraging.” Although the point at which a bee becomes a forager varies, under normal colony conditions, forager bees are workers with an age of more than 3 weeks, and they shift to perform outside tasks, such as collection of water, nectar, pollen, and resin^38, 39^.

Because the time duration of our experiment was shorter than 3 weeks, the experimental duration and bees’ age were almost similar, so that there were no “real foragers” in our experiment. We considered bees that went outside as “forager candidates”; however, they could become real foragers after 3 weeks. In this study, we identified a bee that returned from outside within 10 min before each burst as a “forager candidate”(${\mathrm{F}}_{\mathrm{b}}).$

As we mentioned before, we found that bursts are triggered approximately 10 min after dance events by using ETE between K and D (Fig. 1D). Since dancing is generally performed by bees returning from outside, this means that at least the bees returned from the foraging to the hive that will dance will be in the hive 10 min before the peak of the burst. Therefore, we have designated the bees that returned between 600 and 1000 s (for detecting $\mathrm{P}_\mathrm{b}$ , we used ± 1000 s from the peak of each burst for our analysis. Details in the NMF description section) from the peak of the burst as the foragers of this burst, denoted as ${\mathrm{F}}_{\mathrm{b}}$.

The threshold of 10 min is based on the results in Fig. 1D that the information flow from dance to burst occurs in roughly 10 min, and that the dance is performed by bees returning to the hive from the outside. Moreover, to focus on foraging activities, we had to omit “orientation flights”^16, 17^ that occurred several days before the foraging behavior. To omit orientation flights, the forager candidate’s first day of foraging was defined as the first day on which it has at least six reads of going outside and > 25% of which occurred before 12:00 (based on personal observations, Gene Robinson’s group was aware that in their locality, most orientation flights occur in the afternoon). This criterion for the detection of the orientation flight is similar to that in the previous study by Gene Robinson’s group^18^. Any flight activity on or after this day was defined as foraging activity.

### Detection of waggle dance event

By performing a “waggle dance,” foragers can share directions and the distance to patches of flowers and water sources with other hive mates^30,40,41^. The dancing bees waggle back and forth as they move forward in a straight line and then circle around to repeat the dance. We developed an algorithm for detecting a waggle dance in which the rotation of the bee was used as an indicator of the waggle dance.

The orientation of a bee at time *t*, where ${{n}_{x}}_{t}$ and ${n}_{{y}_{t}}$ denote the respective $x$ and $y$ components of the head direction of each bee, is as follows:

$${n}_{t}={(n}_{{x}_{t}},{n}_{{y}_{t}})$$

If ${n}_{t}\cdot {n}_{t+1}<0$, then the orientation of the bee changed to > 90°. As the first condition was to determine whether a bee was dancing, they used the following parameter: in 5 consecutive seconds, there should be at least four negative scalar products, which implies that, in 5 consecutive seconds, the potential dancer had to change its orientation at least four times to > 90°.

As the second criterion, if ${n}_{t}\times {n}_{t+1} >0$, then the bee made a right turn. When the orientation of the bee alternated between left turn and right turn over 5 consecutive seconds, it was identified as a “potential dance.” We identified a bee that met both conditions as a “dance.” To confirm the success rate of the detection, we attempted to validate the results by watching the videos of the detected dances and by considering the features of the dance (e.g., circling, have followers, and trophallaxis) that were related to dance. We found that, in our tested list of 500 dances, the success rate of the detected dances was 75–82%.

### Detection of dance follow event

Dance followers receive information about the feeding area from dancers^30, 31^. Their existence was observed by von Frisch in 1967^30^. The dance follower tracks the motion of a dancer by keeping its head facing. In this study, we defined a dance follower as a bee that is within 600 pixels (approximately the size of a bee) of the detected dancer when the dancer is dancing and where the direction vector from the follower to the dancer and the vector of the follower’s head direction are always within 90°.

### Detection of a “burst”

We also required a criterion to detect the “bursting” phase. Kleinberg’s burst detection algorithm was used to detect bursting events^21^. This algorithm assumes that the intervals of the events occur independently according to the following exponential distribution:

$$f\left(x\right)=\overline{\lambda }{e}^{-\overline{\lambda }x}$$

Here,$\overline{\lambda }$ is the mean frequency, defined as $N/T$, where *N* is the total number of events over the time series and *T* is the total length of the time series. In addition, $x$ is the interval between consecutive events. Bursts were detected by comparing the expected frequency with the actual event frequency observed within the specific time window.

This algorithm originally required timestamps of the sequence of events in question. However, our data are provided as a time series ${K}_{G}$. Here, we consider ${K}_{G}$ to be a frequency-based sequence, and we must convert our frequency-based data into interval-based data. We simply calculated the inverse of the frequency as the interval between events. For example, a KE value of 1 implies that an event occurs every second. On the other hand, with a KE value of 10, the frequency of an event is considered to be every 0.1 s. Based on this calculation, the total number of events can be calculated. We applied the algorithm after removing the diurnal cycle of ${K}_{G}\left(t\right)$. The removal of the daily cycle was performed by subtracting the time series of KE obtained by applying a moving average with a time window of 12 h from the original KE time series.

We defined the burst level at every time point $t$ of individual events. The burst level was expressed as $bl\left(t\right)$. When $bl\left(t\right)$ was ≥ 1, we considered it as a burst. When the local event frequency at time $t$, shown as $\lambda$, exceeded a certain threshold, the burst level was updated. When ${\lambda }_{t}$ exceeded $\overline{\lambda }{s}^{1}$, the burst level $bl\left(t\right)$ became 1 from 0. In the same manner, if ${\lambda }_{t}$ exceeded $\overline{\lambda }{s}^{2}$, $bl\left(t\right)$ became 2 from 1, and so on. We used $s=2$, so the burst level would increase by 1 when the frequency doubled the previous one.

The algorithm would have detected a large number of bursts whenever the actual frequency of events fluctuated around the boundary between two burst levels, 0 and 1. To alleviate this, we introduced another parameter $\gamma$ into the algorithm. We used $\gamma =1$ (see reference^21^ for details). We defined the “burst period” as a period when the burst level was ≥ 1. We selected these parameter values by observing the time series to ensure that the algorithm detects most of the certain detects we see.

In this study, we explored the bursting behavior detected by Kleinberg’s burst detection algorithm. However, there were also small burst-like activities that were not detected by Kleinberg’s algorithm (depending on the parameters of the algorithm). We speculate that there are some reasons why these small bursts do not grow into larger, global bursts. By examining these small bursting behaviors, we may elucidate a more detailed mechanism for the bursting behavior.

Thinking about the biological significance of these parameters is an interesting idea. While we don't have an immediate answer to this, our proposed computational agent model has an algorithm that is similar to Kleinberg's. This algorithm includes a threshold for agents to become activated (which corresponds to s) and the kinetic energy of the active state compared to those in a quiet state (which corresponds to λ). Since these parameters in the computational model are applied to individual agents, they are not equivalent to the ones in Kleinberg's algorithm. However, we believe that these are biological meanings of those parameters.

### Effective transfer entropy between K, O and D

To examine the relationship of influence (information flow) between the time series of ${K}_{G}$ (K), the number of out-hive bees (O) and the number of waggle dancers (D), we calculated effective transfer entropy (ETE)^23^. ETE is a metric used to quantify the directional flow of information between two interacting systems or variables, which is an improvement of the original transfer entropy (TE) developed by Schreiber^24^. TE from a time series $Y\left(t\right)\left(={y}_{1},{y}_{2},\dots \right)$ to $X\left(t\right)\left(={x}_{1},{x}_{2},\dots \right)$ is given by

$${TE}_{Y \to X} = \mathop \sum \limits_{{}} p\left( {x_{t + 1} ,{\varvec{x}}_{{\varvec{t}}}^{{\left( {\varvec{k}} \right)}} ,{\varvec{y}}_{{\varvec{t}}}^{{\left( {\varvec{l}} \right)}} } \right) \cdot \log \left( {\frac{{p\left( {x_{t + 1} | {\varvec{x}}_{{\varvec{t}}}^{{\left( {\varvec{k}} \right)}} ,{\varvec{y}}_{{\varvec{t}}}^{{\left( {\varvec{l}} \right)}} } \right)}}{{p\left( {x_{t + 1} |{\varvec{x}}_{{\varvec{t}}}^{{\left( {\varvec{k}} \right)}} } \right)}}} \right)$$

where ${{\varvec{x}}}_{{\varvec{t}}}^{\left({\varvec{k}}\right)}=\left({x}_{t},\dots ,{x}_{t-k+1}\right)$, ${{\varvec{y}}}_{{\varvec{t}}}^{\left({\varvec{l}}\right)}=\left({y}_{t},\dots ,{y}_{t-l+1}\right)$, and $p\left( {x|y} \right)$ denotes the conditional probability. It is well known that the estimates of transfer entropy are generally biased owing to small sample effects. To avoid this bias, we calculated the effective transfer entropy (ETE) as follows:

$${ETE}_{X\to Y}={TE}_{X\to Y}-{TE}_{{X}_{\text{Shuffle}}\to Y}$$

where ${X}_{Shuffle}$ describes the randomized time series of X. By shuffling the time series, the time series dependence of X and Y is broken, and we can calculate TE due to the small sample effect. To derive consistent estimates, we repeated the shuffle 300 times and subtracted this average from ${TE}_{X\to Y}$ to obtain the effective transfer entropy.

TE is sensitive to the time window of the data sampling. For instance, estimating TEs using a time series acquired at a sampling rate of 1 s for a time series where information flow exists on a 10-min timescale may not yield correct results. Here, we calculated the ETE between the time series of the number of K, D, O while varying the time window size, ranging from a few minutes to an hour (2, 5, 10, 30, and 60 min). We set the parameter of the estimation of ETE $k$ and $l = 1$. We used the time series of the phase after the entrance was opened for the calculation, because the dance events occurred only at the phase. We used the R package *RTransferEntropy*^42^.

## Classifying bee behaviour by activity and activity timing

To examine when and which bees were initializing and committing to organizing a global burst, we used NMF, which has become a popular decomposition algorithm^25, 26^.

Given a nonnegative matrix $X$, this algorithm finds the nonnegative matrix factors $W$ and $H$, where $W\in {R}^{m \times n}$, $H\in {R}^{r \times n}$, and $r$ is the number of components (known as *rank*), as follows:

$$X \cong WH$$

In practice, $r$ is often chosen such that $r\ll \left(m,n\right)$.

A critical parameter of NMF is the factorization rank $r$ that defines the number of representative bee activities used to approximate the target matrix. The method of setting the value of $r$ is to attempt different values and compute some quality measures and then select the best value. Several approaches are available to decide the optimal value of $r$—for example, Brunet et al. proposed choosing the first value of $r$ for which the cophenetic coefficient starts decreasing^26^. Hutchins et al. suggested choosing the first value where the residual sum of the squares curve presents an inflection point^43^. We decided to use the approach of Brunet et al. in this study. The advantage of this algorithm is that it can factorize the input matrix without destroying the cluster structure of the original matrix. To estimate $r$, we calculated the cophenetic coefficient, changing the value of $r$ from 2 to 10.

In general, $H$ is known as the base (feature) matrix, and $W$ is known as the weight matrix. In the case of our analysis, $X\in {R}^{m \times n}$ implies the ${K}_{i}$ matrix at the bursting phase, as determined by Kleinberg’s algorithm. In addition, $n$ is the number of individual bees, $m$ is the time length of the bursting phase, and the matrix elements are ${K}_{i}$. To extract $X$ from the original ${K}_{i}$ matrix, we explored the timestamps of the peak points for each burst $t{s}_{b}$, where $b$ is the index of the detected bursts. We then defined ${X}_{b}$ as follows:

$${X}_{b}=\left[\begin{array}{c}{k}_{{ts}_{b}-\mathrm{1000,0}} \cdots {k}_{{ts}_{b}+1000,n} \\ \vdots \ddots \vdots \\ {k}_{{ts}_{b}-\mathrm{1000,0}} \cdots {k}_{{ts}_{b}+1000,n}\end{array}\right]$$

Note that we confirmed that the minimum interval of the peaks of the detected bursts was 2538 s. The threshold of 1000 steps were set to be about half of the smallest value (2538 s) of each burst sense, because a threshold longer than 1000 steps may be covered by the falling edge of the previous burst. Also, if the threshold is shorter than 1000 steps, the rising edge of the burst may not be included (i.e., the pioneer bee may not be extracted properly). $H$ describes the representative examples of the time series ${K}_{i}$ (the number of examples [base] depends on $r$). The method starts by randomly initializing the matrices $W$ and $H$, which are iteratively updated to minimize a functional divergence. To estimate the matrices $W$ and $H$, as a local minimum, the NMF algorithm solves the following minimization problem, where $D$ is a loss function based on the Kullback–Leibler divergence:

$$min\left\{ W,H \ge 0 \right\}\left[ D\left(X ,WH\right)\right]$$

There are some types of update functions for solving the prior minimization problem iteratively. In this study, we used the “multiplicative update rules.” The initial entries for $W$ and $H$ were drawn from a uniform distribution. The number of iterations of the update function was 500. The error rate converges sufficiently well at around 500 iterations. We executed it 50 times, changing the initial matrices, and we then selected the solution in which the cost function was minimized.

### Classifying “pioneer bees” using NMF

NMF decomposes the original matrix into a matrix composed of feature vectors and its weight matrix; hence, it is often used as a soft-clustering algorithm. The cluster member was computed as the index of the dominant basis component for each bee. For instance, if the maximum value of the weight vector $w$ ( $= {W}_{\left(,beeZ\right)}$) of bee Z was ${w}_{\left\{i\left(1\le i \le r\right)\right\}}$, bee Z was classified as the *i*th base.

We identified the individual corresponding to the basis vector where the maximum amplitude was observed before the range identified as a burst by the Kleinberg algorithm as a pioneer bee. If the largest value of the recomposed ${K}_{i}$ of bee Z at burst $i$ was smaller than the mean ${X}_{i}$, we did not identify bee Z as a “pioneer bee.”

Such bees were identified for each observed burst. We created the “pioneer-bee binary matrix” $P{M}_{\left\{i,j\right\}}$, where the columns represent each burst index and the rows represent each individual bee index. The matrix element was either 0 or 1—if a bee $i$ was categorized as a “pioneer bee” of the burst $j$, then the matrix element $\left(i,j\right)$ was assigned 1; otherwise, it was 0.

### Dimension reduction of the “pioneer-bee binary matrix”

To examine the similarity between the combination of pioneer bees in different bursts, we conducted a dimensional reduction of $P{M}_{\left\{i,j\right\}}$ using nonmetric multidimensional scaling (nMDS)^33^.

The input data of nMDS is a distance matrix. In this study, we first created the distance matrix from the “pioneer-bee matrix.” We used the Jaccard index for computing the distance of a binary matrix. Next, we reduced the dimension of the distance matrix using nMDS. In the reduced space, the distance between the points exhibited the behavioral similarity of the pioneer bees in each burst. As such, if the same bees were pioneer bees during different bursts, then these bursts are represented close to each other; otherwise, they are presented further apart. We iterated until the stress (the loss function of nMDS) was smaller than 0.2.

### Statistical analysis

We used the Brunner-Munzel test for statistical analysis^44^. This method is an unpaired two-sample test that does not assume normality or equal variances. Since many of our data analyzed in this study could not be assumed to have equal variances or normality, we used this method. The calculations were performed using the lawstat library in R.

## Supplementary Information

Supplementary Information.

## Data Availability

The data that support the findings of this study are available from the corresponding author upon reasonable request.
