# Supplementary material for: Spontaneous and information-induced bursting activities in honeybee hives
Source: Sci Rep. 2023 Jul 7;13:11015. doi: 10.1038/s41598-023-37785-8 (PMC10329038; doi:10.1038/s41598-023-37785-8)
Supplement: Supplementary file 1 — Supplementary Information. [file 41598_2023_37785_MOESM1_ESM.docx]

**Supplement information**

**Title – Spontaneous and Information-induced Bursting Activities in Honeybee Hives**

Itsuki Doi^1^*, Weibing Deng ^2^, Takashi Ikegami^1^

**Affiliations**

^1^ Graduate School of Arts and Sciences, University of Tokyo

^2^ Key Laboratory of Quark and Lepton Physics (MOE) and Institute of Particle Physics, Central China Normal University

**Corresponding author**

Correspondence to: Itsuki Doi1*

**Supplemental Information**

**Computational Model**

We hypothesized that each bee can be activated through direct physical contact with other bees, and that the number of such interactions facilitates its transition from an inactive to an active state (1,2). Some studies have reported the direct physical contact among bees used for their communication (3,4). Hence, we first calculated from the empirical data the number of times the active bees made contact with the inactive bees before they got activated. To compute the probability of getting activated, we divided $K_{i}$ into active and inactive states. Herein, the median value of $K_{i}$ was calculated for each bee, and the active state was defined as the period when $K_{i}$ exceeded twice the median value for more than 2 seconds before returning to the median value (5).

On the time series of binary $K_{i}$ for a particular inactive state, we counted the number of active bees that approached an inactive bee within a given time window τ. **Figure S3.A** shows the frequency of a bee becoming active when the door was closed. For small values of τ, the frequency has a peak at a certain number of hits. As τ became larger, the frequency peak became more gradual. We assumed that bees habituate when they collide more than a certain number of times.

We used an agent-based SIR model (epidemic model) (6,7) to capture the excitation of physical contact between individual bees as follows:

$$I + A \underset{\to}{\alpha} 2A$$

$$A \underset{\to}{\beta} R$$

$$R \underset{\to}{\gamma} I$$

$$I \underset{\to}{\delta} A$$

Each agent is assumed to be in an inactive (I), active (A), or refractory (R) state. An inactive agent is an agent in a normal state that is not yet excited. A An inactive agent transitions to the active state with a probability α, when it comes into contact with an active agent. Active agents start to move much faster than the inactive agents. Active agents change to a refractory state spontaneously with a probability β, and refractory agents return to a normal state again with probability γ. Agents can be spontaneously excited with probability δ. Each burst can be spontaneous or induced by external information. This simulation model does not consider the latter cause.

It's worth noting that this model differs from a basic SIR model in that excited individuals roam about, which increases the probability of collisions with other individuals. This implies that being in an excited state changes behavioral pattern.

**Parameters fixation**


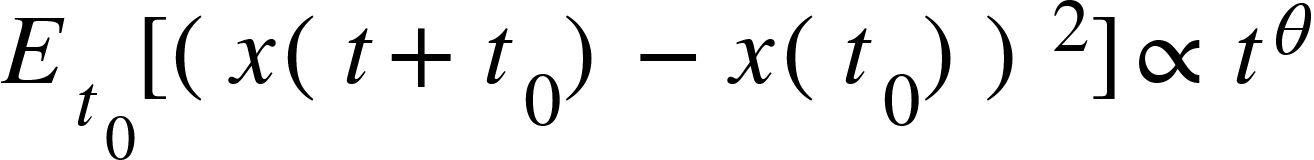
To adjust the parameter values of an agent’s motion, we calculated the displacement speed of the honeybee, where [
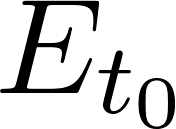
](https://www.codecogs.com/eqnedit.php?latex=E_%7Bt_0%7D%250) represents the average with respect to the variable *t_0_* and *x(t_0_)* is the site of the honeybee at time *t*, as follows:

If $\theta$ is >1, it implies an anomalous diffusion, and $\alpha$ = 1 implies the Gaussian random walk. The average of $\theta$ was 1.2, and the distribution of $\alpha$ obeyed the Gaussian distribution with $\mu$ = 0 and $\varepsilon$ = 1, and the agent occupied a circle with a diameter of 5 (pixels). We calculated the mean of the ratio of the speeds of the inactive and active states using a binarized time series of $K_{i}$ as described above. An active bee appeared to move approximately 10 times (9.8 ± 3.8) faster than an inactive bee.

In this study, an agent's movement followed the Gaussian distribution, with $\mu$ = 0 and $\varepsilon$ = 1. The agent was represented as a circle with a diameter of 5 pixels within the simulation space. The occupancy density *D* of the agent in the environment (the hive) was set to 0.4, which was similar to the actual experimental environmental conditions.

When agent $i$ came in contact with agent $j$, both speeds were reversed. The collision judgment was made when the distance $d_{ij}$ between agent $i$ and agent $j$ became 5 (pixels). An agent’s state and position were initially assigned random values.". In the analysis, the simulation time of one trial was set to 50,000 steps, and the initial 10,000 steps were removed. The results of the subsequent analysis are based on the average values from 10 trials. Figure S4 illustrates an example of the simulated time series of global activities.

Figure S5 depicts the relationship between the parameters ($\alpha$, $\beta$, and γ) as well as the ratio between the mean $K_{G}$ and the maximum value of $K_{G}$. When $\beta$ is <0, the ratio is small—an active bee cannot change refractory bees. Moreover, even when there was an agent in the inactive state, because the active agents overflowed around, the inactive agent turned into an active agent immediately, so the hive was always bursting. When γ is almost 0.00001, an agent cannot exit the refractory state, but when it reaches 0.1, an agent can suddenly change from the refractory state to the inactive state, and it can then become active. For each $\alpha$, the largest ratio was examined. As shown in Figure S6, when $\alpha$ is 0.1, the ratio $max(K_{G})/mean (K_{G})$ is most similar to the empirical ratio.

Here, we fixed $\beta$ at 0.025 and $\gamma$ at 0.0001 because the ratio between the mean $K_{G}$ and the maximum value of $K_{G}$ was most similar to the empirical result ($\hat{r}$ = 10.29 $\pm$ 0.16). To reinforce these selected parameter values, we also calculated the probability as a function of the number of times inactive bees were hit by active bees in the same manner as previously discussed.

Herein, we set $\tau$ = 60 (steps). When $\alpha$ is 0, the probability is basically 0, because an agent goes into the active state spontaneously and no interaction is required. However, when $\alpha$ is >0, the probability that a bee in the inactive state changes to the active state increases when it comes in contact with a bee in the active state. Furthermore, the slope of the function is dependent on $\alpha$. When $\alpha$ is 0.1, the slope of the function may be similar to the empirical data. To measure similarity to the experimental data in more detail, we used a scatter plot of the probability of becoming an active agent. In Figure S7, the results show that the slope of the scatter plot is closest to 1 when $\alpha$ is 0.1. (If the simulated data and the experimental data are completely similar, all data points are on the y = x line.) Based on the estimations in this study, we fixed $\alpha$ at 0.1.

The frequency of the global bursting behavior is related to the excitation probability δ (Fig. S8). To fix $\delta$, we measured the similarity of the distributions of $K_{G}$ and the interburst intervals against the empirical data and the simulation. We used the Jensen–Shannon divergence to measure the similarity among distributions (Fig. S8C). Based on the estimations in this study, we fixed $\delta$ at $5.0 \times{10}^{-5}$.

### Based on these selected parameters, both the probabilistic curve and a bursting curve (the slope of the activation phase) obtained from experimental data can be quantitatively reproduced using the simulation (Fig. S3B, S9). The slopes of modeled activation and deactivation phases of bee activity fit the empirical data well. Therefore, the number of times each bee is in physical contact with other excited bees can trigger global bursting behavior.

With an adequate set of parameters α, β, γ, and δ, we can observe that some agents spontaneously become active and the excitation spreads over the entire simulated hive, resulting in a global burst. However, this simulation does not include foragers or waggle dancers. Thus, the simulation corresponds to a burst when the entrance door is closed.

**Spatial distribution of pioneer bees in the hive and their information flow**

We explored the spatial distribution of pioneer bees in the hive during each burst. We separated pioneer bees into two groups. One group consisted of bees that are foragers and pioneers ($\mathrm{FP}_{b}$ bees, i.e., $F_{b}\wedge P_{b}$), and the other group consisted of bees that are non-foragers but pioneer bees ($\mathrm{NFP}_{b}$ bees, i.e., $\mathrm{NF}_{b}\wedge P_{b}$).

Before the hive entrance was opened (pre-phase), we couldn't confirm a specific distribution within the hive. However, both FP and NFP bees tended to stay near the entrance during the first 1 or 2 days after the hive entrance was opened (post-phase). From the second day onward, the FP bees tended to remain near the entrance, whereas the NFP bees tended to distribute inside the hive. After the third day, the trend in which bees gathered at the entrance more remarkably appeared, and the FP bees stayed near the entrance both before and after the burst, whereas the NFP bees stayed partially near the entrance just before the burst. The tendency to be distributed inside the nest was frequently observed at the decay of the burst (Fig. S10).

We calculated the kinetic energy (KE) for FP, NFP, and non-pioneer bees (NP), as well as the transfer entropy (ETE) among their respective time series (from FP to NFP, FP to NP, NFP to NP, and vice versa). Here, similar to Fig.1D, we calculated the ETE while varying the time window size, ranging from a few seconds to minutes (5, 10, 50, 100, 150, 200, 250, …,600 sec) and the summation of the ETE was calculated for each time bin as follow:

$${ETE}_{Total}=|{ETE}_{FP\to NFP} - {ETE}_{NFP\to FP}|+|{ETE}_{FP\to NP} - {ETE}_{NP\to FP}|+|{ETE}_{NFP\to NP} - {ETE}_{NP\to NFP}|$$

We set the parameters for estimating ETE to $k=1$ and $l=1$. We found that, after the entrance has been open for 3-4 days (namely, around the time when dancers started to appear), there is a tendency for information to flow from FP to both NFP and NP, and from NFP to NP. On the other hand, we found that there is little or no information flow in the opposite direction (e.g., from NP to FP) (Fig.S11).　${ETE}_{FP\to NFP}$ was significantly larger than ${ETE}_{NFP\to FP}$. Note that during the period before dancers appeared (from the opening of the nest to days 1-2), there was no significant information flow between FP and NFP.

**The proportion of pioneer bees classified as pioneers for the first time in each burst**

We assessed the convergence of pioneer bees as the experiment progressed by analysing the proportion of pioneer bee turnovers, in conjunction with the method involving variance in the MDS space, as shown in Fig. 6. The percentage varies depending on the number of previous bursts taken into account, so in this case, we calculated the percentage of unique bees in a specific burst, considering up to four previous bursts as an example (Fig. S12). From this result, we found that the proportion of unique pioneer bees per burst tends to decrease as the experiment progresses (i.e., as the burst ID increases), compared to situations where pioneer bees are selected randomly. This result is consistent with the findings presented in Fig. 6 of the main text.

**Supplemental Figure**


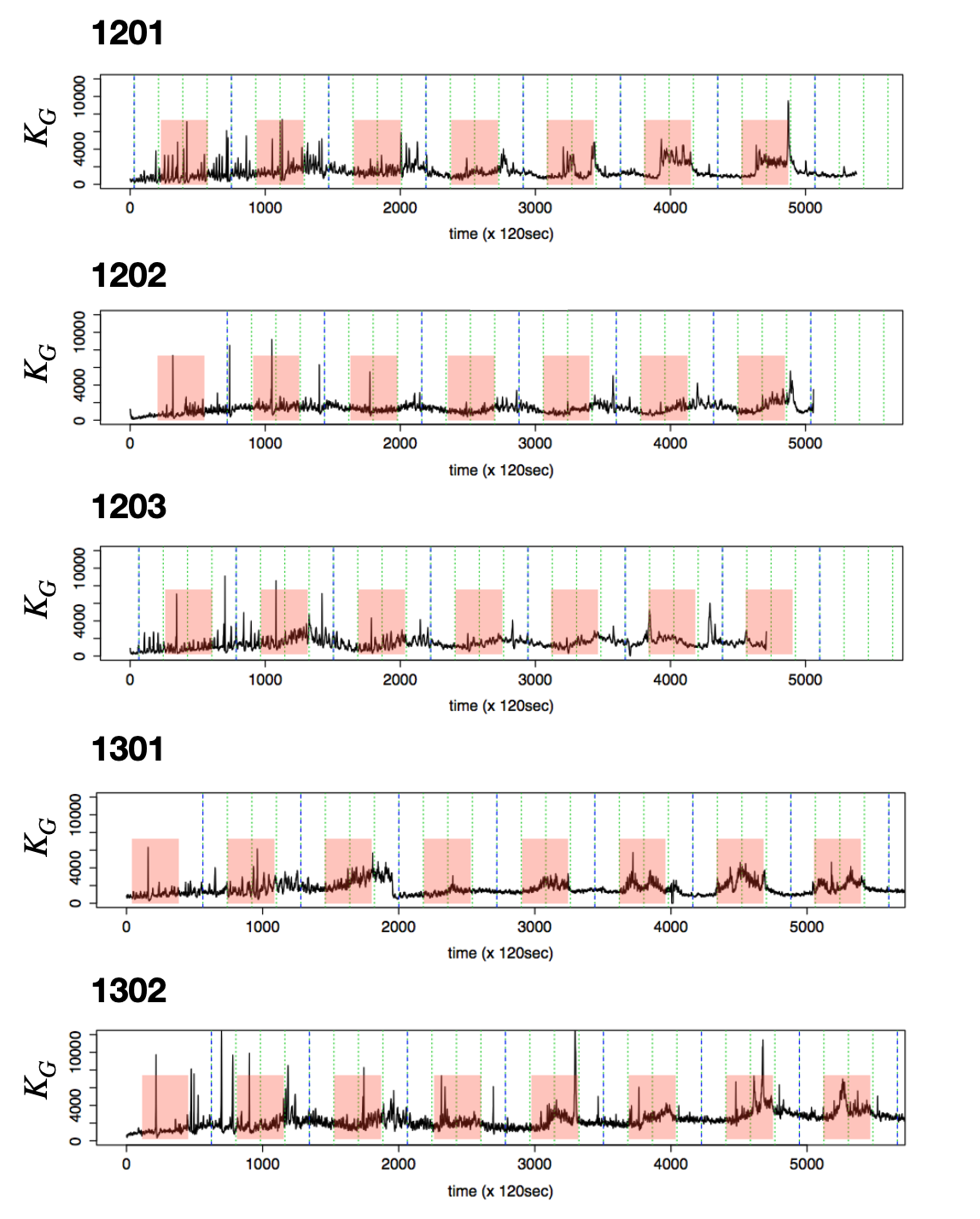


**Figure S1.**

*All time series of* $K_{G}$*.* The parts shaded in orange represent the daytime (4 a.m. to 8 p.m.).　The following figure shows that the bee takes on two very different states, calm and excited.


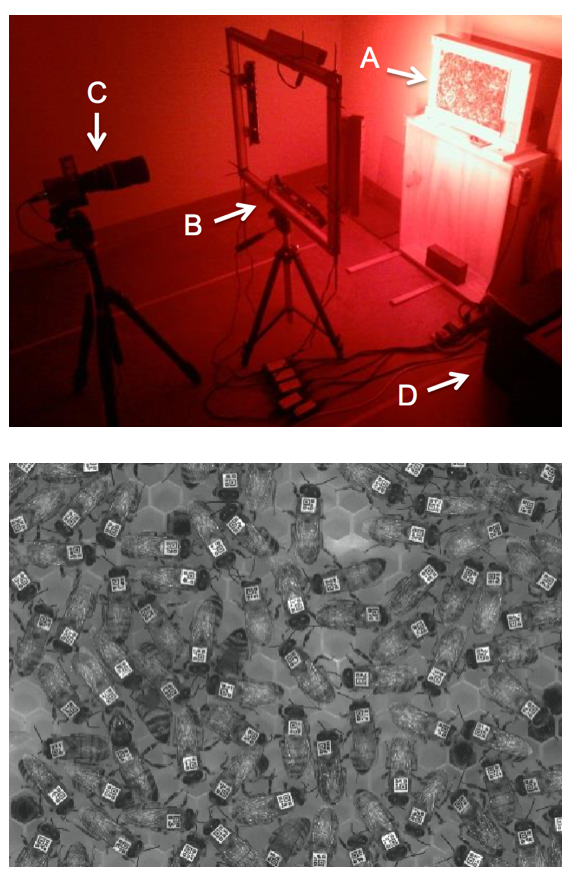


**Figure S2.**

*Experimental setting.* Top: (A) The hive was in a small, glass-walled observation case that held a single honeycomb. The hive was designed to prevent the bees from crawling over each other and ensure maximum exposure to the camera. The hive had an exit to enable the bees to forage outside normally. (B) The hive was illuminated by infrared lights, which bees cannot detect, to ensure natural behavior. (C) The bees were imaged using a high-resolution machine-vision camera (Allied Vision Technologies Prosilica GX6600). It recorded one image per second continuously throughout the experiment. (D) The images were stored on a disk array. Bottom: An example image obtained from this system shows bar-coded bees inside the observation hive. The photos were provided by Tim Gernat.

**Figure S3.**

*The frequency of a bee becoming active following inactivity.* (A). An appropriate number of collisions between bees cause the inactive bee to transition to the active state. (B). The agent-based SIR model can reproduce the similar distribution forms in (A). The dashed lines display the average, and the dotted lines show the standard error. The colored numbers describe τ (Number of active individuals approached within a given time τ)


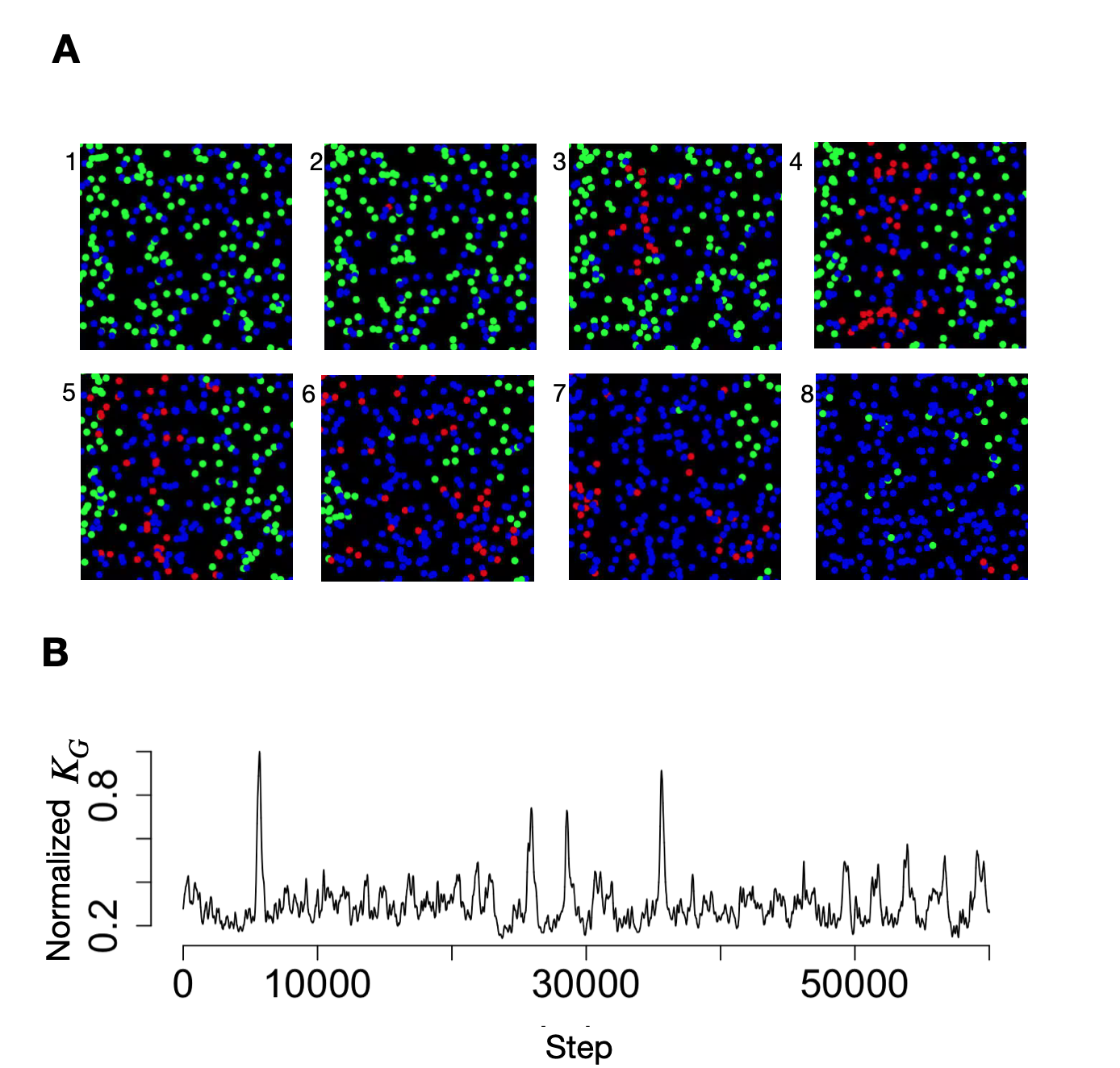


**Figure S4**

*Examples of the simulation A) snapshots of the simulated agents, and B) the time series of* $K_{G}$*.* (A) An inactive bee (blue) transitions to an active state (red).　The time sequence is from the top left to the bottom right. (B) A global burst is induced in the agent model. The value of $\alpha$ is 0.1, $\beta$ is 0.025, and $\gamma$ is 0.0001.


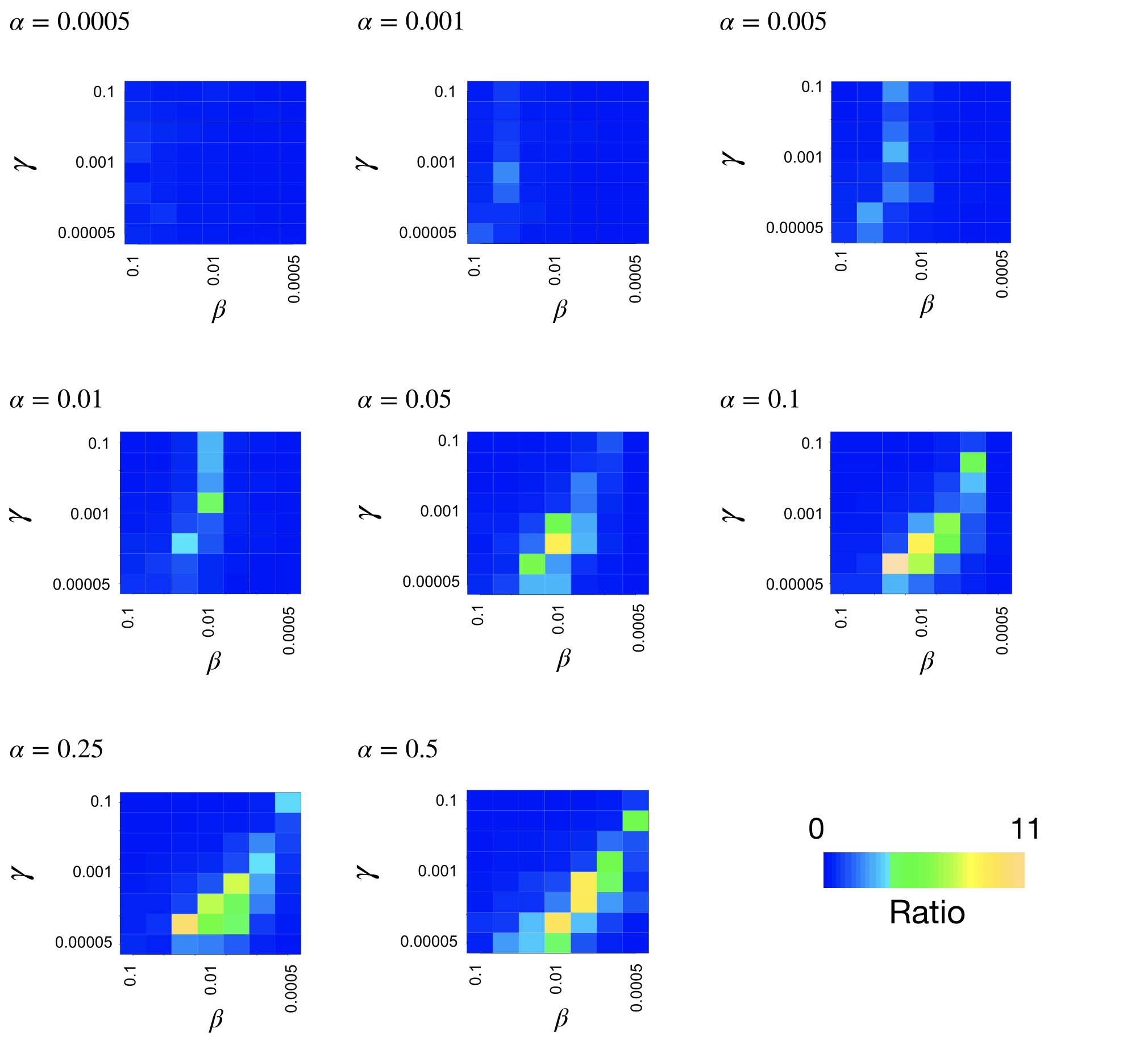


**Figure S5**

*Phase space of the mean to maximum value Kg in terms of* $\alpha$*,*$\beta$*, and* $\gamma.$ The colors describe the ratio between the mean $K_{G}$ and the maximum value of $K_{G}$. See also Fig. S6.


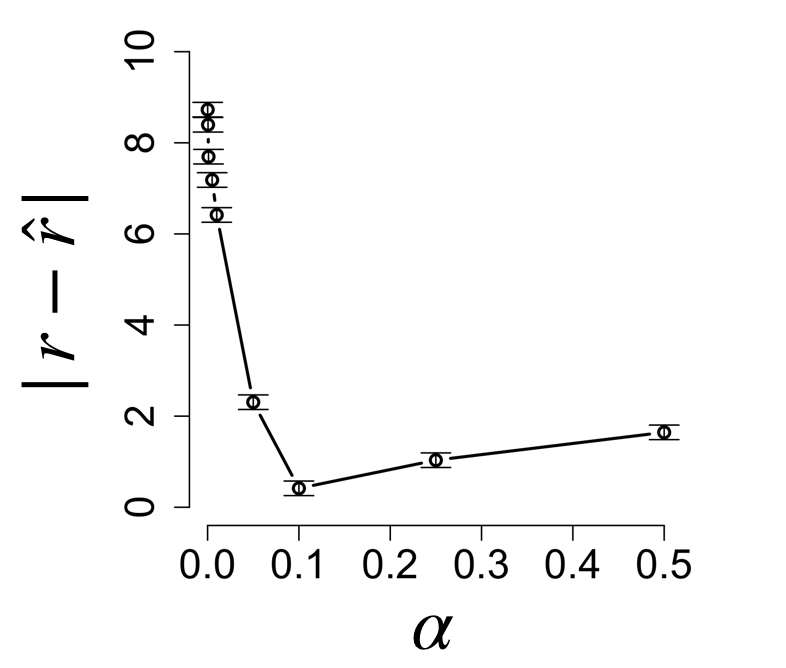


**Figure S6**

*The proximity between the real data and the model is viewed as the ratio of the maximum value of Kg to the variance, with alpha as the parameter.* The *r* value is the maximum ratio between the mean simulated $K_{G}$ and the maximum value of the simulated $K_{G}$. The $\hat{r}$ value is the ratio observed in the empirical data (10.29 $\pm$ 0.16).


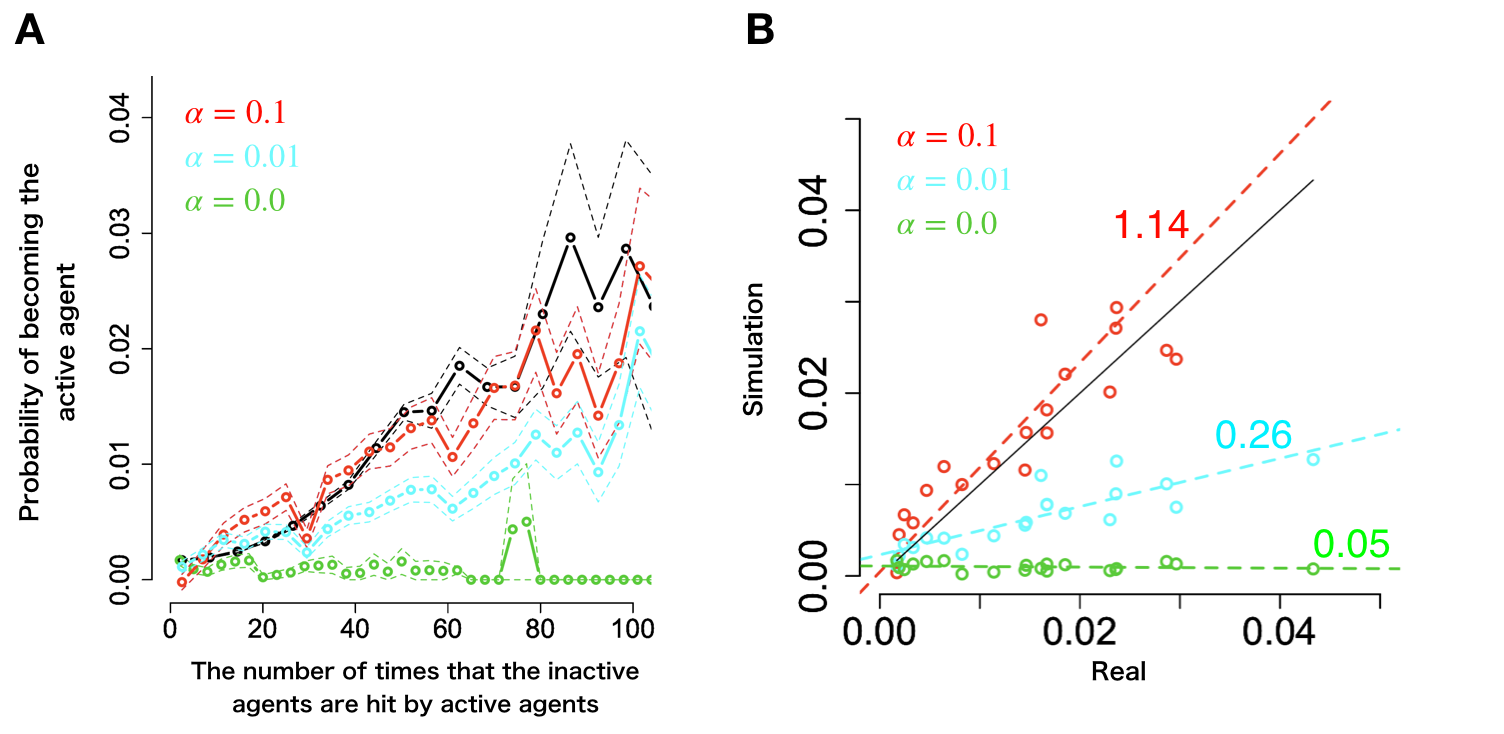


**Figure S7**

*Histogram of the number of times inactive agents hit by active agents and of that changed their state to active: A) empirical data points and B) simulated data points.* (A) The black line describes the empirical data, the red line describes $\alpha$ = 0.1, the blue line describes $\alpha$ = 0.01, and the green line describes $\alpha$ = 0.0. The other parameters were fixed at $\beta$ = 0.025 and $\gamma$ = 0.0001. The dotted lines describe the standard errors. (B) The color description is the same as for Figure S6A. The black solid line describes $y = x$, and the dotted lines are the lines fitted by the linear regression. The value near each fitted line is its slope.

*
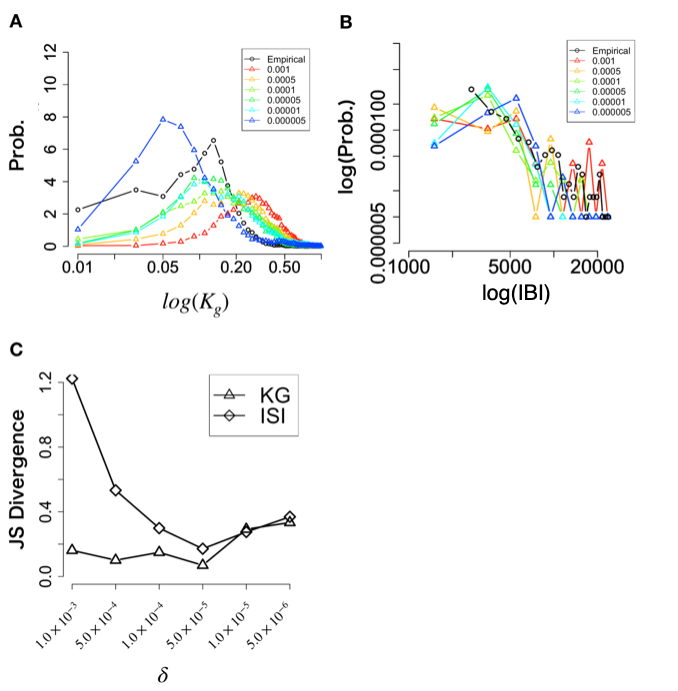
*

**Figure S8**

*Parameter estimation for* $\delta$. The value of $\delta$ is the excitation probability of simulated agents. (A) The distributions of $K_{G}$. The black line describes real honeybees’ $K_{G}$ and the colored lines describe the distributions of the simulated $K_{G}$, and each color represents a different $\delta$. (B) The distributions of the inter burst intervals (IBI). The black line describes the distribution of the IBI of real honeybees during the daytime. The colored lines describe the distribution of the IBI in the simulation, and each color represents a different $\delta$. (C) The Jensen–Shannon divergence between the empirical distribution and simulated distribution. The x-axis is $\delta$., and the y-axis is the Jensen–Shannon divergence.


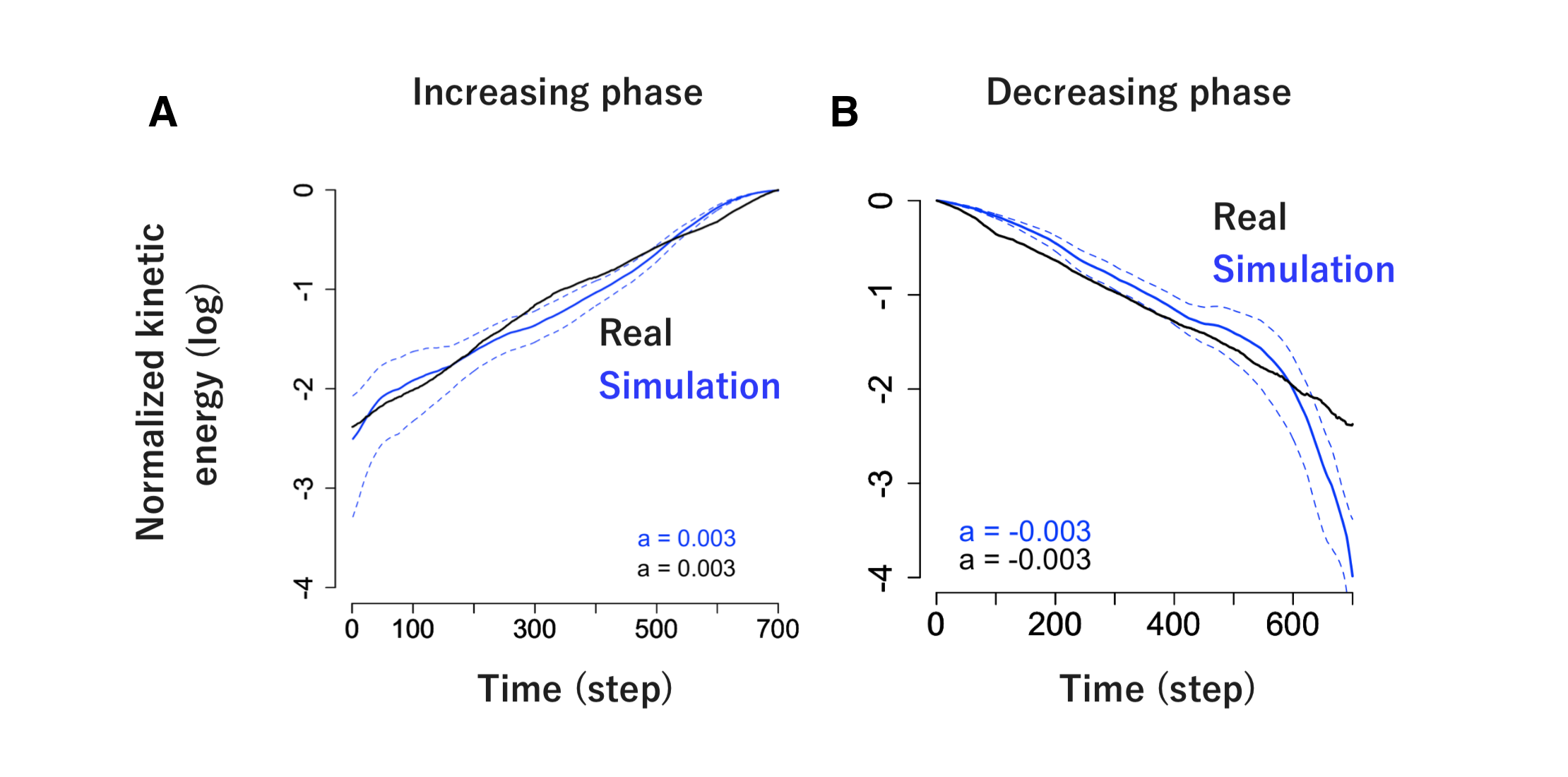
**Figure S9**

*Comparison between the empirical bursts and simulated bursts* *during the increasing (A) and decreasing phases (B) of a burst****.*** The black solid line depicts the average of all the experimental trials. The blue solid line shows the simulated average. The slopes of the regressions are $a$, fitted to the range of the x-axis, i.e., 200–500 (increasing phase) and 100–500 (decreasing phase).

### ****

**Figure S10**

### *The spatial distribution patterns within the hive of FP bees and NFP bees as the average of all trials.* The location of the entrance at the top-right side of each heatmap. “before” columns mean the spatial distribution pattern *before* each burst started. “after” columns mean the spatial distribution pattern after each burst’s peaks. “pre” refers to before the hive entrance was opened, “post” refers to after the hive entrance was opened. Colour indicates relative occupancy, smoothed by kernel density estimation. FP bees tended to locate near the hive entrance, but NFP bees tend to locate in deeper area in the hive.

**Figure S11**

*Effective transfer entropy (ETE) between FP, NFP, and NP after the entrance has been open for 3-4 days.*The black bars describe ETE from FP to NFP, FP to NP and NFP to NP. The light gray bars describe ETE of opposite signals. The p value was determined using the Brunner–Munzel test. * indicate significant difference between ${TE}_{X\to Y}$ and ${TE}_{Y\to X}$on 10% significance levels. Note that the p-value was below 0.1 for all combinations using the Welch's t-test.

**Figure S12**

*The proportion of pioneer bees classified as pioneers for the first time in each burst.* The pink dots represent the proportion of bees that became pioneer bees for the first time in each burst, while the black dots represent the proportion of bees that became pioneer bees for the first time in each burst when pioneer bees were randomly selected (as a negative control). Each line represents the result of linear regression.

**Table S1**

*Detailed information of the experiments.*

| Data name | Experimental date | The number of  bees | The number of detected bursts | Date when the entrance was opened | Mean dance duration (s) | First day of the detected dance event |
| --- | --- | --- | --- | --- | --- | --- |
| 1201 | 2012/7/4 ~ 7/12 | 925 | 73 | 2012/7/5 22:09 | 11.8 ± 0.16 | 2012/7/6 15:38 |
| 1202 | 2012/7/18 ~ 7/24 | 946 | 48 | 2012/7/19 21:26 | 10.6 ± 0.17 | 2012/7/20 16:15 |
| 1203 | 20127/28 ~ 2012/8/3 | 958 | 58 | 2012/7/30 0:34 | 11.0 ± 0.18 | 2012/7/30 16:00 |
| 1301 | 2013/7/3 ~ 2013/7/11 | 1165 | 69 | 2013/7/5 22:24 | 10.8 ± 0.13 | 2013/7/6 11:50 |
| 1302 | 2013/7/16 ~ 7/24 | 965 | 66 | 2013/7/17 21:14 | 9.36 ± 0.06 | 2013/7/18 11:50 |

**References**

1. Davidson, J. D., Arauco-Aliaga, R. P., Crow, S., Gordon, D. M. & Goldman, M. S. Effect of interactions between harvester ants on forager decisions. *Front. Ecol. Evol.* **4**, 115 (2016). [10.3389/fevo.2016.00115](https://doi.org/10.3389/fevo.2016.00115)

2. Gordon, D. M. *Ant encounters in Ant Encounters* (Princeton University Press, 2010)

3. Southwick, E. E. & Moritz, R. F. A. Social synchronization of circadian rhythms of metabolism in honeybees (Apis mellifera). *Physiol. Entomol.* **12**, 209–212 (1987). [10.1111/j.1365-3032.1987.tb00743.x](https://doi.org/10.1111/j.1365-3032.1987.tb00743.x)

4. Korst, P. J. A. M. & Velthuis, H. H. W. The nature of trophallaxis in honeybees. *Ins. Soc.* **29**, 209–221 (1982). [10.1007/BF02228753](https://doi.org/10.1007/BF02228753)

5. Cole, S., Donoghue, T., Gao, R., & Voytek, B. (2019). NeuroDSP: A package forvneural digital signal processing. Journal of Open Source Software, 4(36), 1272. DOI: 10.21105/joss.01272

6. Anderson, R. M. & May, R. M. *Infectious Diseases of Humans: Dynamics and Control* (Oxford University Press, 1992)

7. Anderson, R. M., Discussion. The Kermack-McKendrick epidemic threshold theorem. *B. Math. Biol.* **53**, 1 (1991)
